# Effects of a Modified Exposure Claim for an e-Cigarette on Claim Comprehension, Behavioral Intentions, and Risk Perceptions Among US Adult Tobacco Users and Nonusers: Randomized Experimental Study

**DOI:** 10.2196/85802

**Published:** 2026-04-20

**Authors:** Pierpaolo Magnani, Medy Ehtesham, Alexandre Soulan, Gerd Kallischnigg, Christopher Russell, Steve Roulet

**Affiliations:** 1 Philip Morris International Neuchâtel Switzerland; 2 ARGUS- Statistics and Information Systems in Environment and Health GmbH Berlin Germany; 3 Russell Burnett Research and Consultancy Glasgow United Kingdom

**Keywords:** cigarettes, electronic cigarettes, intentions, public health, risk perceptions, switching, tobacco

## Abstract

**Background:**

Effective communication about the relative risks of cigarettes and e-cigarettes can help increase switching away from cigarettes while minimizing unintended use.

**Objective:**

This study examined comprehension of a proposed modified exposure claim (MEC) about an e-cigarette (IQOS VEEV, the study product [SP]) and the effects of claim exposure on SP use intentions and risk perceptions among adult tobacco users and nonusers.

**Methods:**

Adult smokers with no intention to quit smoking (S-NIQ, n=606), adult smokers with an intention to quit smoking (S-IQ, n=600), adult e-cigarette users (ECU, n=630), adult former smokers (FS, n=619), adult tobacco and nicotine products (TNP) never-users aged 18-24 years (n=648), and adult TNP never-users aged 25 years and older (n=749; total N=3852) participated in a randomized between-groups online experimental study. Participants viewed a marketing brochure for the SP with (test condition) or without (control condition) an embedded MEC. Outcome measures included claim comprehension, intention to use the SP regularly, and perceived health risk to self from using the SP or smoking cigarettes.

**Results:**

Most participants were female (n=2110, 54.8%), had a mean age of 40.2 (SD 14.93) years, and were equally split across the 4 US regions. S-IQ and S-NIQ were long-term, frequent cigarette smokers, while 91.4% (566/619) of FS were long-term quitters. ECU on average used e-cigarettes ≥15.2 times per day, and the majority of them (552/630, 87.6%) had started using e-cigarettes more than 12 months before. Most participants correctly understood the key elements of the claim: the SP produces lower levels of harmful chemicals compared to cigarettes (1818/1926, 94.4%), and switching completely from cigarettes to the SP reduces exposure to harmful chemicals (1832/1926, 95.1%). In both conditions, positive intention to use the SP was high among ECU (control: 238/314, 75.8% vs test: 249/315, 79%; *P*=.33), moderate among S-IQ (control: 127/299, 42.5%; test: 166/299, 55.5%; *P*<.001) and S-NIQ (control: 140/299, 46.8%; test: 166/307, 54.1%; *P*=.07), low among FS (control: 28/306, 9.2%; test: 33/312, 10.6%; *P*=.55), and very low among adult TNP never-users aged 18-24 years (control: 3/330, 0.9%; test: 8/318, 2.5%; *P*=.11), and adult TNP never-users aged 25 years and older (control: 5/373, 1.3% vs test: 12/375, 3.2%; *P*=.09). All groups understood that the SP posed a lower health risk compared to cigarettes. In all groups, claim exposure was associated with significantly lower risk perception of the SP relative to cigarettes (all comparisons, *P*<.001).

**Conclusions:**

The tested MEC has the potential to benefit public health by simultaneously increasing already high levels of SP use intention and reducing SP risk perceptions relative to cigarettes among adult tobacco users while generating low levels of use intention among tobacco nonusers.

## Introduction

Despite a substantial decline in adult smoking prevalence over the past several decades, 28.8 million (11.6% of all) US adults (aged ≥18 years) were current cigarette smokers in 2022 [1]. Between 50%-70% of adults who smoke are projected to die prematurely from smoking-related diseases if they continue to smoke [2,3], losing around 10 years of life relative to adults who never start smoking [3]. The best action that a person who smokes can take to protect and improve health is to completely stop all tobacco and nicotine use. Since many adults continue to smoke, reducing tobacco-related harms among this population is a public health priority.

The United States Food and Drug Administration (FDA) Center for Tobacco Products recognizes that tobacco products exist on a “continuum of risk,” with combusted or “smoked” products, such as cigarettes, posing the greatest health risks, and noncombusted products, such as e-cigarettes and smokeless tobacco, generally considered to be lower risk alternatives for adults who smoke, but not risk-free [4,5]. Therefore, although complete cessation of all tobacco use is optimal, complete switching from cigarettes to a tobacco product that sits lower on the risk continuum has the potential to substantially reduce an individual’s health risks and so represents the second-best option to complete cessation, and one that may be more viable and appealing to adults who are open to stopping smoking but not to stopping all nicotine use.

Absolute and relative risk perceptions are well-established as important predictors of intentions and actions to initiate, continue, and transition between cigarette smoking and e-cigarette use. Adults who smoke and perceive e-cigarettes to be equally or more harmful than cigarettes are significantly less likely to transition from exclusive cigarette smoking to exclusive e-cigarette use [6-8]. Among adults who do switch, the belief that e-cigarettes are less harmful than cigarettes reduces the likelihood of resuming smoking up to 1 year later [7]. In 2018, approximately three-quarters (22.5 million) of US adults who smoked held the belief that e-cigarettes are equally or more harmful than smoking cigarettes, of which only 3.3% completely switched to e-cigarettes in the following year [7]. The authors’ analysis indicated that approximately 1.1 million more adults would likely have switched from cigarettes to e-cigarettes between 2018 and 2019 had they correctly perceived e-cigarettes to be less harmful than cigarettes. Similarly, Persoskie et al [9] estimated that an additional 370,000 adult dual users of cigarettes and e-cigarettes would have completely switched to e-cigarettes between 2013 and 2014 had they correctly perceived e-cigarettes to be less harmful than cigarettes.

Evidence of this significant population-level relationship between lower-risk perceptions of e-cigarettes and adults’ likelihood of adopting and subsequently switching from cigarettes to e-cigarettes, together with evidence that a declining proportion of US adults who smoke believe that e-cigarettes are less harmful than cigarettes, suggests that misperceptions that e-cigarettes pose similar or higher health risks than cigarettes harm public health by maintaining smoking among adults who otherwise may be open to switching. Additionally, with some evidence indicating that this risk misperception is more prevalent among some minority and vulnerable populations [6], it may also contribute to health disparities associated with smoking. Population-level harm may be reduced significantly, therefore, by informing adults who smoke that completely switching to e-cigarettes may substantially lower their health risks.

Marketing e-cigarettes as having “modified” or reduced risks represents one such opportunity available to manufacturers to communicate the absolute and relative risks of using a new e-cigarette product to potential consumers. To market a new tobacco product in the United States, for use to reduce harm or risk of tobacco-related disease, the manufacturer must first submit a modified risk tobacco product (MRTP) application and obtain a marketing granted order from the FDA [10,11]. In deciding on an MRTP order, the FDA evaluates the application for evidence that potential consumers understand the modified risk/exposure claims in the context of the product’s label, labeling, and marketing materials, and the impact that advertising of the new product with the proposed modified risk/exposure claims will have on tobacco use behavior of current tobacco users (eg, adoption, regular use, switching from cigarettes, dual use, and switching back to cigarettes) and nonusers (eg, initiation, reinitiation, and progression to use of more harmful products) [12].

Prospective evidence of the actual behavioral impact of exposure to modified risk/exposure claims about e-cigarettes is limited and difficult to obtain prior to receipt of an MRTP order. In the absence of direct behavioral evidence, data that characterize behavioral intentions and risk perceptions of a new e-cigarette product following exposure to marketing materials with and without presentation of a modified exposure claim (MEC) can inform estimates of the product’s future population use and associated health impact. This study evaluated comprehension of an MEC for a closed-end e-cigarette (the study product [SP]) and tested the effects of exposure to marketing material containing the MEC on adult tobacco users' and nonusers’ intentions to use the SP and the risk perceptions of the SP relative to cigarettes.

## Methods

### Study Design

A randomized, parallel, between-groups, 2-condition experimental study was conducted in 2 parts. In part 1, participants completed a self-administered online survey about their current and past tobacco and nicotine use. Responses were used to classify participants into 1 of 6 mutually exclusive groups according to criteria presented in Multimedia Appendix 1 [13] (described in the “Tobacco Use” section below). In part 2, participants were randomly assigned to 1 of 2 study conditions (control or test). Participants in the test condition viewed a marketing brochure for the SP, which contained an MEC for the SP. Participants in the control condition viewed a brochure for the SP without the claim. Study enrollment began on November 14, 2022; data collection ended on April 14, 2023.

### Study Product

The SP under the brand name of “IQOS VEEV” is an electronic vaping device (also referred to as an e-cigarette or vaping product) that produces an aerosol for inhalation through vaporization of a nicotine-containing liquid. The SP is a closed e-cigarette comprising 2 main components: a replaceable cartridge containing e-liquid and a heating element that connects to a device containing electronics with a rechargeable battery. The marketing brochure presented the device to participants as “IQOS VEEV” and presented the disposable replaceable cartridges as “IQOS VEEV Cartridges.”

### Participants

The study planned to recruit a total of approximately 4320 US-based, English-speaking adults aged 18 years and older who fit the criteria for classification into one of the following six mutually exclusive groups: (1) adult smokers with no intention to quit smoking (S-NIQ), (2) adult smokers with an intention to quit smoking (S-IQ), (3) adult e-cigarette users (ECU), (4)adult former smokers (FS), (5) adult tobacco and nicotine products (TNP) never-users aged 18-24 years inclusive (TNU18-24), and (6) adult TNP never-users aged 25 years or older (TNU25+). In this study, adult smokers were stratified into 2 groups—S-NIQ and S-IQ—given that e-cigarette use intentions may vary by level of intention to quit smoking. Definitions for each group are provided in Multimedia Appendix 1. Classifications were based on participants’ self-reported use of cigarettes, e-cigarettes, and other tobacco products, consistent with guidelines established by the World Health Organization [13]. Excluded individuals were those who (1) did not provide acceptable proof of age; (2) were employed in the fields of market research, public relations, journalism, the tobacco industry, or the health sector; (3) had participated in a clinical study or in research about tobacco or nicotine-containing products in the past 3 months; (4) preferred not to answer the question on sex; (5) started smoking cigarettes/using e-cigarettes within the last 30 days; (6) quit smoking cigarettes/using e-cigarettes within the last 30 days; (7) did not have access to a device appropriate for viewing the study materials; (8) did not acknowledge the informed consent form or did not provide consent to participate; and (9) did not confirm they had the opportunity to review the applicable privacy notices. Stratified randomization was performed using the permuted-block technique to balance the composition of each group on the following three demographic variables: (1) US Census region (US Census Bureau–designated region: Northeast, South, Midwest, and West), (2) sex (male or female), and (3) age (S-NIQ; S-IQ; ECU; and FS aged 21-35 years, 36-50 years, and >51 years; TNU18-24 [18-20 years and 21-24 years]; TNU25+ [25-35 years, 36-50 years, and >51 years]). Randomization of the total sample to study conditions was stratified by group (n=6), US Census region (n=4), sex (n=2), and age (n=3, except for TNU18-24, n=2). This randomization method ensured that an approximately equal number of participants in each group was assigned to each condition (planned n=360).

### Recruitment

Participants were recruited from proprietary databases consisting of people who had indicated an interest in participating in consumer research studies and provided consent to receive invitations to participate. The databases included basic demographic information about potential participants, which was used to create sample frames comprising individuals likely to satisfy study eligibility criteria. Participants were also recruited through retail mall site intercepts and via digital advertising (eg, digital banner advertisements and advertisements on social media platforms). Potential participants received an email invitation to participate in the study, which provided basic information about the study, including the need to be screened for eligibility, the anticipated time to complete the survey, compensation for completion, and a hyperlink that routed to part 1 of the study survey.

### Procedures

Part 1 of the survey involved completion of the screening. Participants who completed part 1 of the survey and satisfied all study eligibility criteria were qualified to continue to part 2 of the survey. Qualified participants were given a link to an appointment scheduling platform where they could schedule an appointment to complete part 2, which involved an interviewer-led interview using a videoconferencing solution, with participants reading and answering some survey questions on their own.

In part 2, participants were age-verified and randomized to the test or control condition. Participants assigned to the test condition viewed a brochure for the SP containing the MEC (Figure S1A in Multimedia Appendix 1). Participants assigned to the control condition viewed the same brochure without the MEC (Figure S1B in Multimedia Appendix 1). Each brochure was a mockup generated specifically for use in this study, in electronic form, and consisted of 3 pages. Page 1 (images of the SP device and packaging containers for SP cartridges in 2 flavor variants [tobacco and menthol]) and page 2 (description of features of the SP) were identical in all respects (ie, content, font size, colors, and positioning) for participants in both conditions.

For participants in the test condition, page 3 displayed an image of the SP with the following MEC: “IQOS VEEV produces significantly fewer and lower levels of harmful chemicals compared to cigarettes. Scientific studies have shown that switching completely from cigarettes to IQOS VEEV significantly reduces your body’s exposure to harmful chemicals. Important information: IQOS VEEV is not risk-free.” For participants in the control condition, the third page displayed the same image of the SP device without the MEC.

Upon opening the assigned brochure, participants were asked by the interviewer to read 1 page at a time, to carefully read all the information on each page, even if the participant did not feel the information applied to them, and to click “next” when they were ready to read the next page. For each page, the interviewer ensured that participants spent sufficient time viewing each page of the brochure before moving to the next page. Images of the assigned brochure were displayed to participants on screen in a manner that was broadly compatible with common devices.

Participants viewed the assigned brochure prior to answering survey questions that measured study outcomes. Questions were displayed in the same fixed order—comprehension, intention to use (ITU) the SP, and risk perceptions—for all participants, as applicable. Participants were not allowed to change their answers after moving to the next question in the survey. Prior to the comprehension assessment, participants were informed that the aim of the assessment was not to test their memory but rather to assess the effectiveness of the brochure at communicating key information about the SP. Accordingly, participants were able to reopen the assigned brochure at any time during the comprehension assessment. Instructional and factual manipulation checks were used to determine participants’ attentiveness during review of the assigned brochure. Given the low proportion of participants who incorrectly answered either the attention check or manipulation check questions, all participants were included in the analysis. Upon survey completion, participants were debriefed to address any misperceptions about the SP that may have arisen from participation in the study. The debriefing script stated that the marketing brochure viewed was for research purposes only and that participants should not assume that using SP is safer than using any other tobacco or nicotine-containing product, such as cigarettes.

### Measures

#### Eligibility

In part 1 of the study, screening questions assessed participants’ eligibility to participate against study inclusion/exclusion criteria.

#### Demographics

Questions assessed participants’ age, sex, race/ethnicity, US state of residence, highest educational attainment, and household income.

#### Tobacco Use

Participants were classified into 1 of 6 mutually exclusive groups based on their responses to questions that assessed their ever use, ever-established/fairly regular use, current use, and frequency/intensity of each of a spectrum of tobacco and nicotine-containing products, including cigarettes, e-cigarettes, oral smokeless tobacco, hookah tobacco, pipe tobacco, and cigars/cigarillos. Intention to quit smoking was measured by two questions based on the Stages of Change model developed by Prochaska and DiClemente [14]: (1) “Are you seriously considering quitting smoking within the next 6 months?” (yes/no) and (2) “Are you planning to quit smoking within the next 30 days?” (yes/no). This approach assessed participants’ readiness to quit smoking according to the distinct stages outlined in the model.

### Comprehension of the MEC

Comprehension of the key elements of the MEC was assessed by 4 questions (provided in Multimedia Appendix 1). Questions assessed participants’ understanding that (1) the SP produces significantly lower levels of harmful chemicals compared to cigarettes, (2) exposure to harmful chemicals is significantly reduced when switching completely from cigarettes to the SP, (3) in order for a person to reduce his/her body’s exposure to harmful chemicals, he/she should stop smoking cigarettes and only use the SP, and (4) using the SP would be associated with some risk (ie, would not be risk-free). For each question, participants selected a single response from 5 options (questions 1, 2, and 3) or 2 options (question 4; yes/no). Each question had only 1 correct response, and all questions included a “don’t know/not sure” option. The comprehension question closely followed the guidance recommended by the FDA for label comprehension studies. In both the test and control conditions, 2 questions additionally assessed participants’ understanding that the SP (1) is a vaping product/electronic cigarette and (2) contains nicotine [15].

### ITU the SP Regularly

ITU the SP regularly was measured by a single question: “If you try IQOS VEEV and like it, how likely or unlikely would you be to use IQOS VEEV regularly?” Participants selected a single response from a 6-point Likert scale (1=Definitely not, 2=Very unlikely, 3=Somewhat unlikely, 4=Somewhat likely, 5=Very likely, and 6=Definitely). Participants were also able to select “don’t know.” This question was displayed only to participants who had previously indicated they were “Somewhat likely,” “Very likely,” or “Definitely” would try IQOS VEEV in response to the question: “Based on what you know about IQOS VEEV, how likely or unlikely are you to try IQOS VEEV?”

### Risk Perception

Risk perception was measured using the Assessment of Behavioral Outcomes Related to Tobacco and Nicotine Containing Products (ABOUT)-Perceived Risk Instrument [16-18]. The ABOUT-Perceived Risk Instrument is a psychometrically validated, self-reported measurement instrument that assesses the perceived risks associated with the use of tobacco and/or nicotine containing products (eg, cigarettes, e-cigarettes, heated tobacco products, and nicotine replacement therapy products), and the perceived risks associated with the past use of tobacco and/or nicotine containing products (ie, cessation). The instrument assesses perceived risks to the individual (their personal risk) in 2 domains (perceived health risk to self and perceived addiction risk) using 2 unidimensional scales. The Perceived Health Risk Scale measures the perceived negative impact of product use on the user’s physical health, starting with minor, immediate manifestations of health risk (eg, having sores of the mouth or throat) to more serious long-term manifestations (eg, having lung cancer).

In this study, the 9-item short version of the Perceived Health Risk Scale measured perceived health risk to self from use of (1) cigarettes, (2) the SP, (3) e-cigarettes, and (4) cessation [18]. Responses were measured on a 5-point Likert scale (0=no risk, 1=low risk, 2=moderate risk, 3=high risk, and 4=very high risk) [16,17]. Participants could also select an “I don’t know” option for each item. “Don’t know” responses were interpreted as missing values and replaced by the mean response to other items by the same participant. Missing value imputation was only carried out for participants with ≤50% missing values. A small number of risk perception assessments were not applicable to some groups and/or tobacco-related products and so were not assessed (ie, adult TNP never-users were not assessed on risks that are perceived to remain after smoking cessation).

### Statistical Analysis

All analyses were performed with SAS software (version 9.4; SAS Institute Inc; 2021). All analyses were specified a priori. Categorical outcome measures are described by the total number of participants in each group with nonmissing data and by the number and proportion of participants in each group endorsing each response option. Continuous outcome measures are described by the number of participants in each group with nonmissing score values, and by appropriate measures of central tendency and dispersion. Participant disposition is summarized. Descriptive statistics are reported for all demographic and participant characteristics (age, sex, race, ethnicity, highest educational attainment, US Census region of residence, and household income), overall and stratified by group and study condition.

Analyses of claim comprehension, ITU the SP, and risk perception outcomes included all participants who completed the survey. The number and proportion of participants in the test condition who selected the predefined correct answer and each incorrect answer in response to each measure of claim comprehension are reported separately for each group.

The number and proportion of participants who selected each response option on the 6-point ordinal scale measure of “intention to use the SP regularly” (1=Definitely not, to 6=Definitely) is reported for each group within each condition. For each group, the effect of the study condition on the ITU score between the test and control conditions was tested by the Mann-Whitney *U* test. To account for multiple comparisons, *P* values were calculated based on a Bonferroni-adjusted type I level of 0.0167. “Positive intention” to use the SP regularly was operationalized in this study as the proportion of participants who indicated they would be “Very likely” or “Definitely” to use the SP regularly (ie, response option 5 or 6 on the ITU scale). The number and proportion (unadjusted 95% CIs) of participants who expressed a positive ITU the SP regularly are reported separately for each group within each condition. For each group, a 2 × 2 chi-square test tested the association between study conditions (test vs control) and the level of ITU the SP regularly (positive intention vs nonpositive intention).

A total score was calculated for the Perceived Health Risk to Self Scale and transformed into a Rasch-derived measure on a 0 to 100 scale (where 0=no risk and 100=very high risk) in accordance with instrument scoring procedures [16]. Descriptive statistics and unadjusted 95% CIs of the perceived health risk scores are reported for each group within each condition separately for the SP, cigarettes, e-cigarettes, and cessation. The difference between transformed Rasch-derived scores for perceived health risk to self for cigarettes and the SP (calculated as SP score minus cigarettes score) was calculated for each condition. The difference between cigarettes and SP (SP minus cigarettes) was calculated for each condition and compared by group in both the test and control conditions using a 2-tailed, unpaired *t* test at the Bonferroni-adjusted type I level of 0.0167 to account for multiple comparisons.

### Ethical Considerations

This study was approved by the Sterling Institutional Review Board (10288). All participants provided informed consent prior to enrollment in the study. The informed consent form included a description and purpose of the study, the duration and compensation for participating, as well as information that the findings may be published externally. Participants who completed parts 1 and 2 of the study were compensated US $50. All data analyses were conducted on deidentified datasets, with no individual data disclosed.

## Results

### Disposition

**Figure 1:**
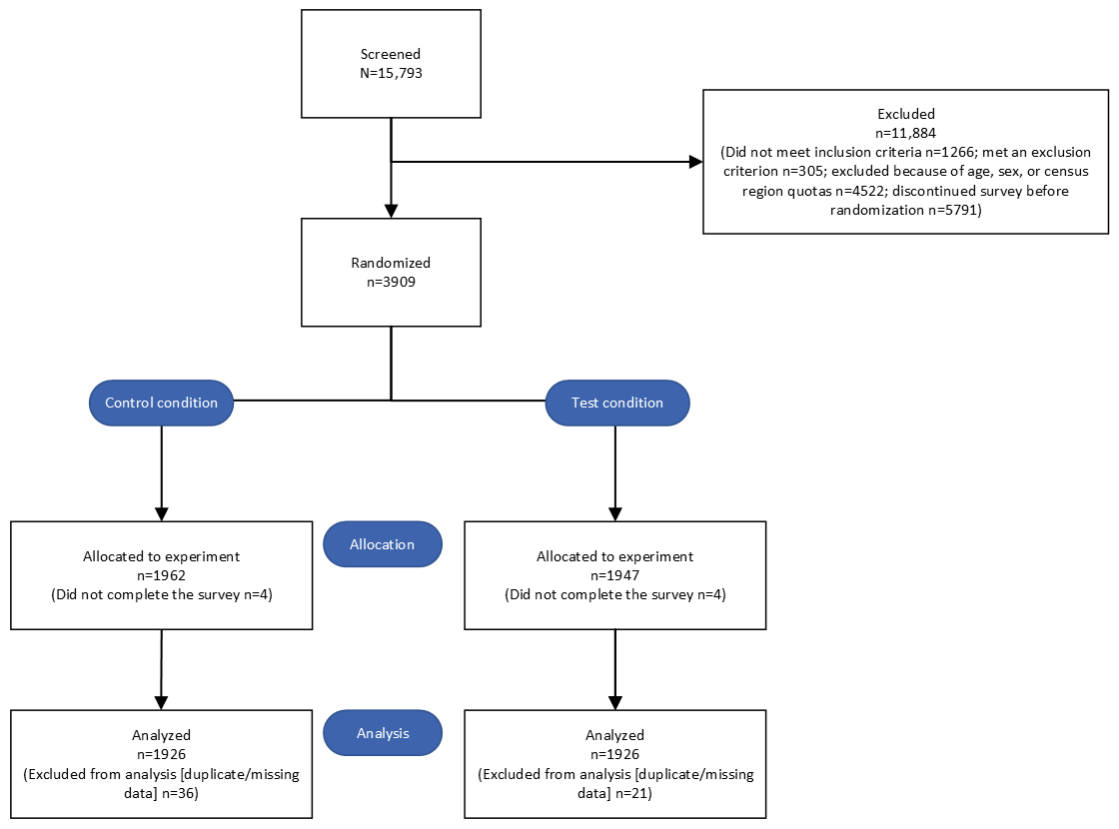
CONSORT (Consolidated Standards of Reporting Trials) flow diagram for a US-based, randomized, online experimental study evaluating a modified exposure claim for the study product (an e-cigarette). The diagram shows the number of participants screened, excluded, randomized, and included in analyses across test and control conditions. Study enrollment and data collection occurred between November 14, 2022, and April 14, 2023.

The CONSORT (Consolidated Standards of Reporting Trials) guidelines were used to ensure the reporting of this experimental study (Multimedia Appendix 2) [19]. Figure 1 presents the flow diagram of the study.

A total of 15,793 individuals were screened, of whom 11,884 (75.2%) were excluded (reasons for exclusion are presented in Figure S2 in Multimedia Appendix 1). A total of 1962 participants and 1947 participants were randomized to the control and test conditions, respectively. In total, 1926 participants in each condition completed the study survey. Thus, the final study sample included 3852 participants across 6 mutually exclusive groups, as shown in Table 1.

**Table 1:** Random assignment of participant groups to study conditions. Participants were classified into 6 mutually exclusive tobacco use groups—adult smokers with no intention to quit (S-NIQ), adult smokers intending to quit (S-IQ), adult e-cigarette users (ECU), adult former smokers (FS), and tobacco and nicotine products never-users aged 18-24 years (TNU18-24) and ≥25 years (TNU25+). Participants were randomized to view a product brochure either with (test) or without (control) a modified exposure claim for the study product (an e-cigarette).

Group	Control (n=1926), n (%)	Test (n=1926), n (%)	Total (n=3852), n (%)
S-NIQ	299 (15.5)	307 (15.9)	606 (15.7)
S-IQ	301 (15.6)	299 (15.5)	600 (15.6)
ECU	315 (16.4)	315 (16.4)	630 (16.4)
FS	307 (15.9)	312 (16.2)	619 (16.1)
TNU18-24	330 (17.1)	318 (16.5)	648 (16.8)
TNU25+	374 (19.4)	375 (19.5)	749 (19.4)

### Demographic and Tobacco Use Characteristics

Participants predominantly identified as female (n=2110, 54.8%), White (n=2732, 70.9%), and not Hispanic (n=3227, 83.8%; Table 2). The final sample was approximately evenly distributed across the 4 US Census regions. The mean age was 40.3 (SD 14.97) years and 40.0 (SD 14.97) years in the test and control conditions, respectively. Participant distributions across age group, sex, US Census region, highest educational attainment, race, and ethnicity were comparable between the test and control conditions.

**Table 2:** Participant demographic characteristics, stratified by study condition. Participants were randomized to view a product brochure either with (test) or without (control) a modified exposure claim for the study product (an e-cigarette). Characteristics are stratified by study condition.

Assessment		Control (n=1926)	Test (n=1926)	Total (n=3852)
Age (years), mean (SD)		40.0 (14.89)	40.3 (14.97)	40.2 (14.93)
**Age group, n (%)**				
	18-20 years	178 (9.2)	172 (8.9)	350 (9.1)
	21-24 years	228 (11.8)	207 (10.7)	435 (11.3)
	25-35 years	431 (22.4)	442 (22.9)	873 (22.7)
	36-50 years	572 (29.7)	583 (30.3)	1155 (30.0)
	51+ years	517 (26.8)	522 (27.1)	1039 (27.0)
**Sex, n (%)**				
	Male	872 (45.3)	870 (45.2)	1742 (45.2)
	Female	1054 (54.7)	1056 (54.8)	2110 (54.8)
**US Census region, n (%)**				
	Midwest	440 (22.8)	434 (22.5)	874 (22.7)
	Northeast	480 (24.9)	481 (25.0)	961 (24.9)
	South	487 (25.3)	489 (25.4)	976 (25.3)
	West	519 (26.9)	522 (27.1)	1041 (27.0)
**Ethnicity, n (%)**				
	Hispanic or Latino	255 (13.2)	253 (13.1)	508 (13.2)
	Not Hispanic or Latino	1614 (83.8)	1613 (83.7)	3227 (83.8)
	Prefer not to say	56 (2.9)	60 (3.1)	116 (3.0)
	Missing	1 (0.1)	0 (0.0)	1 (0.0)
**Race^a^, n (%)**				
	American Indian or Alaska Native	36 (1.9)	44 (2.3)	80 (2.1)
	Asian	149 (7.7)	137 (7.1)	286 (7.4)
	Black or African American	335 (17.4)	370 (19.2)	705 (18.3)
	Native Hawaiian or Other Pacific Islander	7 (0.4)	10 (0.5)	17 (0.4)
	White	1385 (71.9)	1347 (69.9)	2732 (70.9)
	Prefer not to say	89 (4.6)	92 (4.8)	181 (4.7)
	Missing	1 (0.1)	0 (0.0)	1 (0.0)
**Education attainment, n (%)**				
	Less than high school	11 (0.6)	7 (0.4)	18 (0.5)
	Some high school or General Education Development	74 (3.8)	76 (3.9)	150 (3.9)
	High school graduate	252 (13.1)	236 (12.3)	488 (12.7)
	Some college	670 (34.8)	657 (34.1)	1327 (34.4)
	College graduate	690 (35.8)	704 (36.6)	1394 (36.2)
	Advanced degree	223 (11.6)	241 (12.5)	464 (12.0)
	Prefer not to say	5 (0.3)	5 (0.3)	10 (0.3)
	Missing	1 (0.1)	0 (0.0)	1 (0.0)

^a^Answers are not mutually exclusive. The total percentage may be higher than 100%.

More than two-thirds (834/1206, 69.2%) of S-IQ and S-NIQ in the test and control conditions smoked cigarettes “daily,” with the remaining (372/1206, 30.8%) smoking cigarettes “occasionally” (Table S2 in Multimedia Appendix 1). S-IQ and S-NIQ who reported smoking cigarettes “occasionally” smoked cigarettes on approximately 10 days in the past month. In each of the test and control conditions, S-IQ and S-NIQ smoked approximately 13 cigarettes on average on each smoking day. Almost all S-IQ and S-NIQ (1167/1206, 96.8%) started smoking cigarettes more than 12 months ago. These results indicate that S-IQ and S-NIQ were predominantly long-term, frequent cigarette smokers. The vast majority (566/619, 91.4%) of FS were long-term quitters, having last smoked a cigarette more than 12 months ago. ECU used e-cigarettes approximately 16 times per day, on average, with most (552/630, 87.6%) having started using e-cigarettes more than 12 months ago.

### Claim Comprehension

Comprehension of key elements of the MEC was high overall, highest among TNU18-24, and lowest among ECU. The difference between the lowest and highest rate of comprehension was 11.5% on each of the 4 elements of the claim across groups. Following reading of the claim, the majority of participants in each group correctly understood that (1) the SP produces lower levels of harmful chemicals compared to cigarettes (correctly understood range: 284/315, 90.2% [ECU] to 313/318, 98.4% [TNU18-24]), (2) exposure to harmful chemicals is significantly reduced when switching completely from cigarettes to the SP (correctly understood range: 291/315, 92.4% [ECU] to 310/318, 97.5% [TNU18-24]), (3) in order for a person to reduce his/her body’s exposure to harmful chemicals, he/she should stop smoking cigarettes and only use the SP (correctly understood range: 262/315, 83.2% [ECU] to 300/318, 94.3% [TNU18-24]), and (4) there are some risks associated with using the SP (ie, using the SP would not be risk-free; correctly understood range: 265/315, 84.1% [ECU] to 304/318, 95.6% [TNU18-24]; Table 3). In each study condition, most participants in each group also correctly understood that the SP is a vaping product/electronic cigarette (control: 1843/1925, 95.7%; test: 1830/1926, 95%) that contains nicotine (control: 1907/1925, 99.1%; test: 1886/1926, 97.9%; Table S1 in Multimedia Appendix 1).

**Table 3:** Comprehension of key elements of the modified exposure claim.

Assessment			S-NIQ^a^ (n^b^=307), n (%)		S-IQ^c^ (n^b^=299), n (%)		ECU^d^ (n^b^=315), n (%)		FS^e^ (n^b^=312), n (%)		TNU18-24^f^ (n^b^=318), n (%)		TNU25+^g^ (n^b^=375), n (%)		Total (n^b^=1926), n (%)
**Understanding that IQOS VEEV** **produces lower levels of harmful chemicals compared to cigarettes:** **“According to the material, what should Kelly say when asked which of the following statement best describes the amount of harmful chemicals produced by IQOS VEEV compared to cigarettes? Should Kelly say that...”**															
	IQOS VEEV produces higher levels of harmful chemicals than cigarettes	5 (1.6)		1 (0.3)		5 (1.6)		1 (0.3)		1 (0.3)		6 (1.6)		19 (1.0)	
	IQOS VEEV produces the same levels of harmful chemicals as cigarettes	6 (2.0)		10 (3.3)		13 (4.1)		5 (1.6)		4 (1.3)		6 (1.6)		44 (2.3)	
	IQOS VEEV produces lower levels of harmful chemicals than cigarettes (correct)	288 (93.8)		278 (93.0)		284 (90.2)		298 (95.5)		313 (98.4)		357 (95.2)		1818 (94.4)	
	IQOS VEEV does not produce harmful chemicals	8 (2.6)		7 (2.3)		12 (3.8)		8 (2.6)		0 (0.0)		4 (1.1)		39 (2.0)	
	Don’t know/not sure	0 (0.0)		3 (1.0)		1 (0.3)		0 (0.0)		0 (0.0)		2 (0.5)		6 (0.3)	
**Understanding that the exposure to harmful chemicals is significantly reduced when switching completely from cigarettes to IQOS VEEV:** **“According to the material, what should Jennifer say when asked which of the following statements best describes the exposure to harmful chemicals when switching completely from cigarettes to IQOS VEEV? Should Jennifer say that the exposure to harmful chemicals...”**															
	Is significantly increased	4 (1.3)		2 (0.7)		4 (1.3)		0 (0.0)		1 (0.3)		6 (1.6)		17 (0.9)	
	Is significantly reduced (correct)	287 (93.5)		282 (94.3)		291 (92.4)		302 (96.8)		310 (97.5)		360 (96.0)		1832 (95.1)	
	Is eliminated	6 (2.0)		4 (1.3)		8 (2.5)		3 (1.0)		0 (0.0)		1 (0.3)		22 (1.1)	
	Is the same as continuing to smoke cigarettes	9 (2.9)		9 (3.0)		12 (3.8)		6 (1.9)		6 (1.9)		8 (2.1)		50 (2.6)	
	Don’t know/not sure	1 (0.3)		2 (0.7)		0 (0.0)		1 (0.3)		1 (0.3)		0 (0.0)		5 (0.3)	
**Understanding that the exposure to harmful chemicals is significantly reduced when switching completely from cigarettes to IQOS VEEV:** **“The material states that the exposure to harmful chemicals is significantly reduced when switching completely from cigarettes to IQOS VEEV. What should Greg, who is currently smoking cigarettes, do to reduce his body’s exposure to harmful chemicals? Should Greg...”**															
	Continue to smoke the same number of cigarettes and use IQOS VEEV	1 (0.3)		0 (0.0)		1 (0.3)		0 (0.0)		0 (0.0)		0 (0.0)		2 (0.1)	
	Reduce the number of cigarettes and use IQOS VEEV	29 (9.4)		28 (9.4)		50 (15.9)		26 (8.3)		18 (5.7)		23 (6.1)		174 (9.0)	
	Stop smoking cigarettes and only use IQOS VEEV (correct)	274 (89.3)		271 (90.6)		262 (83.2)		285 (91.3)		300 (94.3)		348 (92.8)		1740 (90.3)	
	Increase the number of cigarettes and use IQOS VEEV	2 (0.7)		0 (0.0)		0 (0.0)		0 (0.0)		0 (0.0)		2 (0.5)		4 (0.2)	
	Don’t know/not sure	1 (0.3)		0 (0.0)		2 (0.6)		1 (0.3)		0 (0.0)		2 (0.5)		6 (0.3)	
**Any risk associated with using IQOS VEEV:** **“According to the material, is there any risk associated with using IQOS VEEV?”**															
	Yes (correct)	275 (89.6)		264 (88.3)		265 (84.1)		292 (93.6)		304 (95.6)		349 (93.1)		1749 (90.8)	
	No	28 (9.1)		31 (10.4)		44 (14.0)		19 (6.1)		11 (3.5)		18 (4.8)		151 (7.8)	
	Don’t know/not sure	4 (1.3)		4 (1.3)		6 (1.9)		1 (0.3)		3 (0.9)		8 (2.1)		26 (1.3)	

^a^S-NIQ: adult smokers with no intention to quit.

^b^Number of participants who answered all questions required for the evaluation of this end point and were asked the respective question depending on their assigned group.

^c^S-IQ: adult smokers with an intention to quit.

^d^ECU: adult e-cigarette users.

^e^FS: adult former smokers.

^f^TNU18-24: tobacco and nicotine products never-users aged 18-24 years.

^g^TNU25+: tobacco and nicotine products never-users aged 25 years and older.

### ITU the SP Regularly

When examined on the 6-point ITU scale, ITU the SP regularly was significantly higher in the test (versus control) condition among 2 of the 6 groups: S-IQ and TNU18-24 (both *P*<.001; Table 4). S-IQ rated their ITU the SP regularly higher than the scale midpoint (rank 3.5) in both the control (mean rank 3.6, SD 1.81) and test (mean rank 4.2, SD 1.58) conditions. In contrast, although it is statistically significantly different between conditions, TNU18-24 expressed an ITU the SP regularly that was close to the scale minimum score (rank 1.0) in both the control (mean rank 1.1, SD 0.50) and test (mean rank 1.3, SD 0.88) conditions.

**Table 4:** Intention to use (ITU) the study product regularly, stratified by study condition among adult smokers with no intention to quit (S-NIQ), adult smokers intending to quit (S-IQ), and adult e-cigarette users (ECU) study groups.

			S-NIQ							S-IQ							ECU				
			Control (n^a^=299)		Test (n^a^=307)		*P* value		Control (n^a^=299)			Test (n^a^=299)		*P* value		Control (n^a^=314)			Test (n^a^=315)		*P* value
**ITU rating^b^**																					
	Definitely not, n (%; 95% CI)	64 (21.4; 16.8-26.5)		48 (15.6; 11.7-20.2)		N/A^c^		86 (28.8; 23.6-34.3)			40 (13.4; 9.7-17.8)		N/A		23 (7.3; 4.6-10.8)			20 (6.3; 3.9-9.7)		N/A	
	Very unlikely, n (%; 95% CI)	6 (2.0; 0.7-4.4)		2 (0.7; 0.0-2.4)		N/A		2 (0.7; 0.0-2.4)			4 (1.3; 0.3-3.4)		N/A		5 (1.6; 0.5-3.7)			2 (0.6; 0.0-2.3)		N/A	
	Somewhat unlikely, n (%; 95% CI)	10 (3.3; 1.6-6.1)		11 (3.6; 1.8-6.4)		N/A		11 (3.7; 1.8-6.5)			10 (3.3; 1.6-6.1)		N/A		6 (1.9; 0.7-4.2)			6 (1.9; 0.7-4.1)		N/A	
	Somewhat likely, n (%; 95% CI)	79 (26.4; 21.5-31.9)		80 (26.1; 21.2-31.4)		N/A		73 (24.4; 19.6-29.7)			79 (26.4; 21.5-31.9)		N/A		42 (13.4; 9.8-17.7)			38 (12.1; 8.6-16.2)		N/A	
	Very likely, n (%; 95% CI)	104 (34.8; 29.3-40.5)		112 (36.5; 31.0-42.2)		N/A		94 (31.4; 26.2-37.1)			122 (40.8; 35.1-46.7)		N/A		145 (46.2; 40.5-51.9)			147 (46.7; 41.0-52.4)		N/A	
	Definitely, n (%; 95% CI)	36 (12.0; 8.5-16.3)		54 (17.6; 13.4-22.4)		N/A		33 (11.0; 7.7-15.2)			44 (14.7; 10.9-19.3)		N/A		93 (29.6; 24.6-35.1)			102 (32.4; 27.2-37.9)		N/A	
Mean rank sum, mean (SD; 95% CI)			3.9 (1.69; 3.7-4.1)		4.2 (1.58; 4.0-4.4)		.02^d^		3.6 (1.81; 3.4-3.8)			4.2 (1.50; 4.1-4.4)		<.001^d^		4.8 (1.33; 4.6-4.9)			4.9 (1.26; 4.8-5.0)		.30^d^
Positive ITU^e^, n (%; 95% CI)			140 (46.8; 41.0-52.7)		166 (54.1; 48.3-59.8)		.07^f^		127 (42.5; 36.8-48.3)			166 (55.5; 49.6-61.3)		<.001^f^		238 (75.8; 70.6-80.5)			249 (79.0; 74.1-83.5)		.33^f^

^a^Number of participants who answered all the questions required for the evaluation of this end point.

^b^Question asked: “If you try IQOS VEEV and like it, how likely or unlikely are you to use IQOS VEEV regularly?”

^c^N/A: not applicable.

^d^Bonferroni adjusted type I level of 0.0167. *P* values were based on the Mann-Whitney *U* test calculated for 6-item Likert scales.

^e^The percentage of participants with “Definitely” or “Very likely” ITU.

^f^Chi-square 2-tailed test of independence between test vs control conditions.

When ratings on the 6-point ITU scale were dichotomized, positive ITU the SP (ie, proportion of participants selecting “Very likely” or “Definitely”) was significantly higher for S-IQ only (control: 127/299, 42.5% vs test: 166/299, 55.5%; *P*<.001; Table 4). In both conditions, positive ITU the SP regularly was highest among ECU (control: 238/314, 75.8% vs test: 249/315, 79%; *P*=.33) followed by S-NIQ (control: 140/299, 46.8% vs test: 166/307, 54.1%; *P*=.07) and S-IQ (control: 127/299, 42.5%; *P*<.001 vs test: 166/299, 55.5%; *P*<.001). Positive ITU the SP regularly was lower among FS (control: 28/306, 9.2% vs test: 33/312, 10.6%; *P*=.55) and very low among TNU18-24 (control: 3/330, 0.9% vs test: 8/318, 2.5%; *P*=.11) and TNU25+ (control: 5/373, 1.3% vs test: 12/375, 3.2%; *P*=.09; Table 5).

**Table 5:** Intention to use (ITU) the study product regularly, stratified by study condition—adult former smokers (FS), tobacco and nicotine products never-users aged 18-24 years (TNU18-24), and tobacco and nicotine products never-users aged 25 years and older (TNU25+) study groups.

Statistics		FS				TNU18-24				TNU25+			
		Control (n^a^=306)	Test (n^a^=312)	*P* value	Control (n^a^=330)		Test (n^a^=318)	*P* value	Control (n^a^=373)		Test (n^a^=375)	*P* value	
**ITU rating^b^**													
	Definitely not, n (%; 95% CI)	228 (74.5; 69.2-79.3)	219 (70.2; 64.7-75.3)	N/A^c^	323 (97.9; 95.6-99.2)		288 (90.6; 86.8-93.6)	N/A	345 (92.5; 89.3-95.0)		337 (89.9; 86.3-92.8)	N/A	
	Very unlikely, n (%; 95% CI)	4 (1.3; 0.3-3.4)	9 (2.9; 1.3-5.5)	N/A	1 (0.3; 0.0-1.7)		7 (2.2; 0.8-4.5)	N/A	1 (0.3; 0.0-1.5)		2 (0.5; 0.0-2.0)	N/A	
	Somewhat unlikely, n (%; 95% CI)	10 (3.3; 1.5-6.0)	8 (2.6; 1.1-5.0)	N/A	2 (0.6; 0.0-2.2)		3 (0.9; 0.1-2.8)	N/A	6 (1.6; 0.5-3.5)		6 (1.6; 0.5-3.5)	N/A	
	Somewhat likely, n (%; 95% CI)	36 (11.8; 8.3-16.0)	43 (13.8; 10.1-18.2)	N/A	1 (0.3; 0.0-1.7)		12 (3.8; 1.9-6.5)	N/A	16 (4.3; 2.4-6.9)		18 (4.8; 2.8-7.5)	N/A	
	Very likely, n (%; 95% CI)	25 (8.2; 5.3-11.9)	23 (7.4; 4.7-10.9)	N/A	1 (0.3; 0.0-1.7)		7 (2.2; 0.8-4.5)	N/A	4 (1.1; 0.2-2.8)		10 (2.7; 1.2-4.9)	N/A	
	Definitely, n (%; 95% CI)	3 (1.0; 0.2-2.9)	10 (3.2; 1.5-5.9)	N/A	2 (0.6; 0.0-2.2)		1 (0.3; 0.0-1.8)	N/A	1 (0.3; 0.0-1.5)		2 (0.5; 0.0-2.0)	N/A	
Mean rank sum, mean (SD; 95% CI)		1.8 (1.45; 1.6-2.0)	1.9 (1.57; 1.8-2.1)	.23^d^	1.1 (0.50; 1.0-1.1)		1.3 (0.88; 1.2-1.4)	<.001^d^	1.2 (0.80; 1.1-1.3)		1.3 (0.98; 1.2-1.4)	.19^d^	
Positive ITU^e^, n (%; 95% CI)		28 (9.2; 6.1-13.0)	33 (10.6; 7.3-14.6)	.55^f^	3 (0.9; 0.1-2.7)		8 (2.5; 1.0-4.9)	.11^f^	5 (1.3; 0.4-3.2)		12 (3.2; 1.6-5.6)	.09^f^	

^a^Number of participants who answered all the questions required for the evaluation of this end point.

^b^Question asked: “If you try IQOS VEEV and like it, how likely or unlikely are you to use IQOS VEEV regularly?”

^c^N/A: not applicable.

^d^Bonferroni adjusted type I level of 0.0167. *P* values were based on the Mann-Whitney *U* test calculated for 6-item Likert scales.

^e^The percentage of participants with “Definitely” or “Very likely” ITU.

^f^Chi-square 2-tailed test of independence between test vs control conditions.

### Perceived Health Risk to Self

Overall, participants in the test condition perceived smoking cigarettes to have the highest health risk to self (mean 68.9, SD 14.67), followed by e-cigarettes (mean 53.5, SD 17.80), the SP (mean 48.2, SD 17.61), and cessation (mean 32.8, SD 17.49; Table 6). This order was observed in all groups. Again, groups consisting of FS and adult TNP never-users (FS, TNU18-24, and TNU25+) perceived health risks to self from using the SP, using e-cigarettes, and smoking cigarettes to be higher compared to groups consisting of adult current tobacco users (ECU, S-NIQ, and S-IQ). Perceived health risk to self from cessation was again similar across all groups. Perceived health risk to self among participants in the control condition followed a similar pattern. Overall, participants in the control condition perceived the health risk to self from using the SP (mean 54.4, SD 19.78) to be lower than smoking cigarettes (mean 66.1, SD 16.16), comparable to using e-cigarettes (mean 55.8, SD 19.61), and higher than cessation of all tobacco use (mean 35.9, SD 17.74).

**Table 6:** Perceived health risks to self from using each tobacco product, stratified by participant group and study condition.

Group/product		Control, mean (95% CI)^a^	Test, mean (95% CI)^a^	
**S-NIQ^b^**				
	Participants, n^c^	299	307	
	Cigarettes	57.9 (55.9-59.9)	64.8 (62.9-66.6)	
	e-Cigarettes	47.7 (45.7-49.8)	48.1 (46.0-50.2)	
	SP	45.6 (43.4-47.7)	42.6 (40.7-44.4)	
	TNP cessation	33.6 (31.6-35.5)	32.0 (29.9-34.1)	
**S-IQ^d^**				
	Participants, n^c^	301	298	
	Cigarettes	62.7 (60.8-64.5)	66.9 (65.4-68.4)	
	e-Cigarettes	52.0 (49.8-54.2)	49.7 (47.9-51.6)	
	SP	50.6 (48.6-52.7)	43.3 (41.6-45.0)	
	TNP cessation	37.2 (35.1-39.2)	33.3 (31.3-35.2)	
**ECU^e^**				
	Participants, n^c^	312	315	
	Cigarettes	62.0 (60.1-63.8)	65.6 (63.7-67.5)	
	e-Cigarettes	45.7 (43.6-47.8)	47.3 (45.2-49.3)	
	SP	43.9 (41.7-46.0)	42.0 (40.1-44.0)	
	TNP cessation	35.3 (33.3-37.4)	32.6 (30.5-34.7)	
**FS^f^**				
	Participants, n^c^	306	312	
	Cigarettes	66.5 (64.9-68.0)	68.2 (66.7-69.7)	
	e-Cigarettes	57.5 (55.3-59.7)	53.9 (52.1-55.6)	
	SP	56.6 (54.5-58.7)	49.4 (47.5-51.2)	
	TNP cessation	37.6 (35.6-39.6)	33.2 (31.4-35.0)	
**TNU18-24^g^**				
	Participants, n^c^	329	318	
	Cigarettes	72.5 (71.1-73.9)	71.9 (70.7-73.1)	
	e-Cigarettes	65.2 (63.4-67.0)	59.3 (57.7-61.0)	
	SP	63.9 (62.1-65.7)	54.3 (52.5-56.0)	
	TNP cessation	N/A^h^	N/A	
**TNU25+^i^**				
	Participants, n^c^	374	372	
	Cigarettes	73.0 (71.6-74.4)	74.7 (73.3-76.0)	
	e-Cigarettes	63.8 (61.9-65.6)	60.6 (58.7-62.5)	
	SP	63.1 (61.2-65.0)	55.9 (53.9-57.9)	
	TNP cessation	N/A	N/A	
**Total**				
	Participants, n^c^	1926	1926	
	Cigarettes	66.1 (65.4-66.8)	68.9 (68.2-69.5)	
	e-Cigarettes	55.8 (54.9-56.7)	53.5 (52.6-54.3)	
	SP	54.4 (53.5-55.3)	48.2 (47.4-49.0)	
	TNP cessation	35.9 (34.9-36.9)	32.8 (31.8-33.8)	

^a^Unadjusted 95% CIs.

^b^S-NIQ: adult smokers with no intention to quit.

^c^Number of participants who answered all questions required for the evaluation of the end point.

^d^S-IQ: adult smokers with an intention to quit.

^e^ECU: adult e-cigarette users.

^f^FS: adult former smokers.

^g^TNU18-24: tobacco and nicotine products never-users aged 18-24 years.

^h^N/A: not applicable.

^i^TNU25+: tobacco and nicotine products never-users aged 25 years and older.

This ordering of product use behaviors from highest (cigarettes) to lowest (cessation) health risk to self was observed in all groups. Groups consisting of adult former users and adult TNP never-users (FS, TNU18-24, and TNU25+) perceived the health risk to self from using the SP, using e-cigarettes, and smoking cigarettes to be higher compared to groups consisting of current tobacco users (ECU, S-NIQ, and S-IQ). Perceived health risks to self from cessation were similar across all groups.

**Figure 2:**
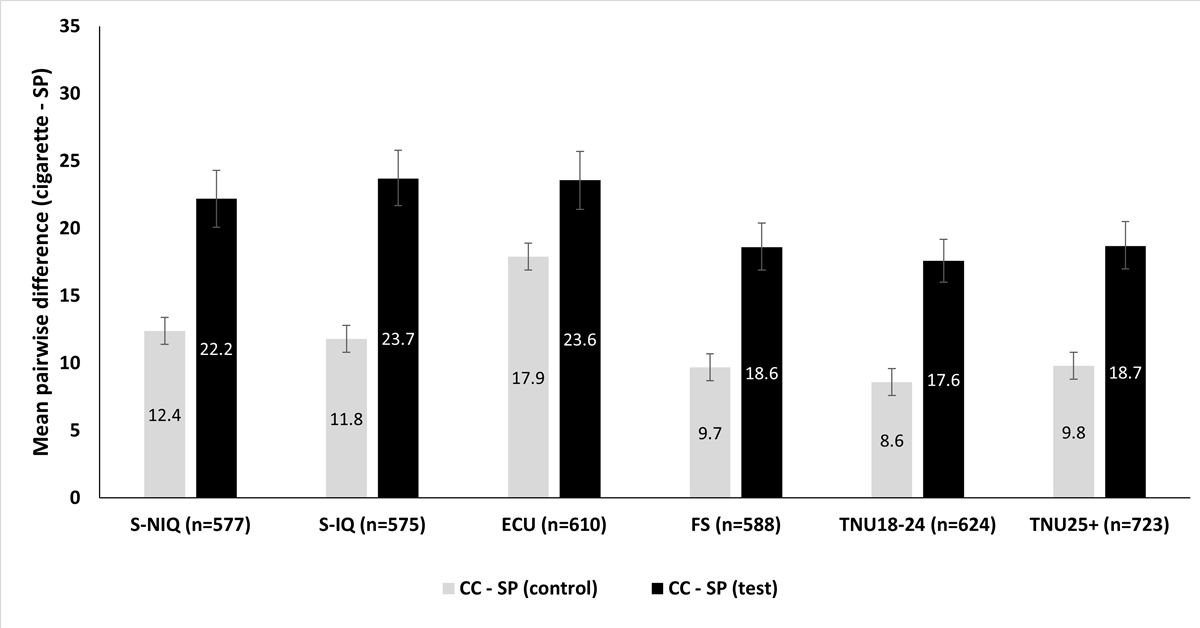
Difference scores for perceived health risk to self from smoking cigarettes versus using the study product (SP). Participants were randomized to view a product brochure either with (test) or without (control) a modified exposure claim. Perceived risk was measured using the Assessment of Behavioral Outcomes Related to Tobacco and Nicotine Containing Products-Perceived Health Risk Scale and transformed to a Rasch score. All *P* values are <0.001. “n” represents the sum of test and control conditions (CC). ECU: adult e-cigarette users; FS: adult former smokers; S-IQ: adult smokers with an intention to quit; S-NIQ: adult smokers with no intention to quit; TNU18-24: tobacco and nicotine products never-users aged 18-24 years; TNU25+: tobacco and nicotine products never-users aged 25 years and older.

Within each group, exposure to the claim (versus no claim) was associated with a significantly larger negative mean difference score for perceived risk to self from using the SP relative to smoking cigarettes (all comparisons, *P*<.001). Mean difference scores for perceived health risk to self (ie, the mean score for smoking cigarettes minus the mean score for using the SP) ranged from 8.6% (TNU18-24) to 17.9% (ECU) in the control condition, and from 17.6% (TNU18-24) to 23.7% (S-IQ) in the test condition (Figure 2). Overall, exposure to the claim increased perceived risk to self of cigarettes and decreased perceived risk to self of the SP, leading to a larger negative mean difference score.

## Discussion

### Principal Findings

e-Cigarettes typically expose users to substantially fewer and lower levels of toxicants compared to cigarettes, which likely translates into lower health risks [20]. Yet, most adults who smoke are unaware and instead believe e-cigarettes to be equally or more harmful than cigarettes [7]. Communicating e-cigarettes’ lower exposure profile could help to correct smokers’ misperceptions and, in turn, increase their likelihood of adopting and switching to e-cigarettes. However, an MEC could harm public health if it leads the public to believe that an e-cigarette’s lower exposure means its use would pose no risk to health. For these reasons, it is important that MECs about e-cigarettes are correctly understood and that the effects of claim exposure on e-cigarette behavioral intentions among tobacco users and nonusers are assessed. This study assessed comprehension of a scientifically substantiated claim, embedded within a mocked-up marketing brochure for the SP, that switching completely from cigarettes to the SP (an e-cigarette) reduces the body’s exposure to harmful chemicals. The effects of this MEC on SP behavioral intentions and risk perceptions among both adult tobacco users and nonusers were also evaluated.

Results indicated high comprehension of key elements of the claim among all groups, with comprehension exceeding the threshold of 80% that is generally accepted for label comprehension studies for all 4 claim communication objectives [21]. The vast majority of adult tobacco users and nonusers understood that the SP produces lower levels of harmful chemicals compared to cigarettes, but also that exposure to lower levels of harmful chemicals does not mean that using the SP would pose no risk. Additionally, the vast majority of adults who smoke cigarettes—the population for whom the switching message is most relevant—understood that exposure to harmful chemicals is significantly reduced when switching completely from cigarettes to the SP (566/606, 93.4%), and understood that a person who smokes would need to stop smoking cigarettes and only use the SP (ie, switch completely from cigarettes to the SP) to achieve this exposure reduction (545/606, 89.9%). These high levels of comprehension closely resemble those elicited by exposure to a very similarly worded MEC for similar e-cigarette products [21], providing evidence to suggest the tested claim may be comparably well understood in the context of a variety of e-cigarette brands and marketing materials.

A small minority (57/606, 9.4%) of adult smokers with and without an intention to quit cigarettes understood that a person who smokes could reduce exposure to harmful chemicals by reducing the number of cigarettes this person smokes while using the SP. This response is incorrect because this is not what the claim stated. However, this response may not reflect participants’ interpretation of the claim, but rather may reflect their preexisting beliefs, their personal experiences of the health benefits of reducing smoking, and/or their awareness of scientific evidence that suggests reducing smoking without cessation can benefit health [22-25] and can lead to subsequent cessation [26-28].

Exposure to a marketing brochure for the SP both with and without the MEC generated moderate to high levels of positive ITU the SP on a regular basis among adults who currently smoke and/or use e-cigarettes while simultaneously generating low ITU the SP among former smokers and very low levels of positive use intention among young and older adults who have never used, or never regularly used, any tobacco or nicotine product. Results showed that claim exposure was associated with significantly higher levels of positive ITU the SP regularly in only 1 of the 6 groups: adult smokers who intend to quit cigarettes. The absolute increase in the proportion of S-IQ who reported an ITU the SP following exposure to the claim was large (+13% versus without the claim), suggesting the SP would likely interest a larger proportion of adult smokers who intend to quit smoking if marketed with the MEC. In contrast, the absolute increase in the proportion of adult tobacco nonusers (FS, TNU18-24, and TNU25+) who reported an ITU the SP following exposure to the claim was small. The overall level of positive ITU suggests that marketing the SP with or without the MEC would be unlikely to lead to any sizeable use among tobacco nonusers, particularly among adult tobacco and nicotine never-users (TNU18-24 and TNU25+). Other studies have reported that young adult nonsmokers’ interest in using snus and e-cigarettes was similarly unaffected by exposure to modified risk messages for these products, particularly when the message’s focus on “reducing risks by switching from smoking” made the products and the message feel less relevant to them as nonsmokers [29,30].

Consistent with this study’s finding that significantly more adult smokers may adopt and use the SP as an alternative to smoking cigarettes when informed that completely switching to the SP would significantly reduce exposure to harmful chemicals, several studies have similarly reported the potential switching benefits of presenting comparative risk messages to adults who smoke. Messages that emphasize the potential benefits of switching completely from cigarettes to e-cigarettes, such as reduced exposure to harmful chemicals, have been found to increase smokers’ intentions to switch completely from cigarettes to e-cigarettes [31,32]. In contrast, messages emphasizing uncertainty about e-cigarette risks (eg, focusing on unknown long-term effects) can reinforce misperceptions that e-cigarettes may be equally or more harmful than cigarettes, potentially deterring smokers from switching [33,34]. This aligns with findings from risk communication research showing that clear, actionable messages about relative risks are generally more effective for promoting harm-reducing behavior change compared to messages that emphasize scientific uncertainty or unknown risks [35,36].

Perceptions of the health risks posed by the different tobacco products and use behaviors in this study are generally aligned with the FDA’s ordering of these products and use behaviors along the “continuum of risk.” Tobacco users and nonusers perceived that continuing/starting to smoke cigarettes would pose the greatest risk to their health, while they perceived the lowest risk would be achieved through the cessation of all TNP use. The health risks of using the SP specifically or e-cigarettes more generally were perceived to fall between smoking cigarettes and complete cessation. Tobacco users also perceived cigarettes, e-cigarettes, and the SP to be riskier than did tobacco nonusers. Findings suggest the tested marketing material with MEC elicited a significantly larger difference between the perceived health risk from smoking cigarettes versus using the SP, indicating the greater effectiveness of the claim (versus no claim) in reducing risk perceptions of the SP relative to cigarettes. Given the evidence that millions more adult smokers would by now have switched completely to e-cigarettes had they correctly held lower risk perceptions of e-cigarettes relative to cigarettes [7,9], marketing the SP with the claim may be significantly more effective than “no claim” in increasing switch intentions. However, prospective, randomized studies would be needed to understand the effects of claim exposure on actual switching behavior and any mediating role of earlier switch intentions attributable to claim exposure.

Study findings should be interpreted in the context of several limitations. This study tested the effects of brief exposure to a single, online instance of marketing material containing a single MEC at a single point in time. It is possible that the use intentions and risk perceptions of the SP may change, strengthen, or weaken with repeated exposure to the same claim presented within the same or different forms of marketing materials over longer periods of time. The study did not assess actual use of the SP or any other tobacco product following exposure to the claim. No conclusions can be drawn, therefore, about the actual behavioral effects of advertising materials or of any mediation effects of this relationship by SP use intentions or risk perceptions. Exposure to actual physical products and marketing materials with claims in real-world retail settings may also elicit use intentions and risk perceptions of the SP that differ from those elicited by this study’s online presentation of 2-dimensional product-related imagery. Prospective observational and interventional studies can address these limitations. In particular, regulatory assessments of the public health impact of a MEC would benefit from longitudinal examination of (1) the stability of use intentions over time following a single exposure to the claim, (2) the effects of increasing the frequency of claim exposure, (3) the effects of exposure to different claims on both behavioral intentions and actual tobacco product use and switching behavior, and (4) the extent to which SP use intentions predict subsequent use behavior. Lastly, sampling from research panels may limit the generalizability of results to the general US adult population. For example, this study recruited a higher proportion of participants who had completed at least some college education (3185/3852, 82.7%) than is found in the general US adult population (63.3%) [37], which may have yielded higher levels of claim comprehension than may be found in the general population.

### Conclusion

Adult TNP users and nonusers understood the claim that switching completely from cigarettes to the SP significantly reduces exposure to harmful chemicals. Viewing marketing materials for the SP with and without the claim simultaneously generated high levels of use intention among adult tobacco users and low levels of use intention among TNP nonusers. Results suggest that marketing the SP with (versus without) the MEC may increase use intentions and reduce risk perceptions of the SP relative to cigarettes among adults who smoke, both of which are known promoters of cigarette to e-cigarette switching behavior.

## Data Availability

We are committed to transparency and will consider any requests for underlying data, provided that such data may be shared under applicable laws.

## Appendix

Multimedia Appendix 1 Participant group definitions, marketing materials, claim comprehension results, disposition, use characteristics, and modified exposure claim comprehension measures.

Multimedia Appendix 2 CONSORT checklist.

## Abbreviations

ABOUT: Assessment of Behavioral Outcomes Related to Tobacco and Nicotine Containing Products
CONSORT: Consolidated Standards of Reporting Trials
ECU: adult e-cigarette users
FDA: United States Food and Drug Administration
FS: adult former smokers
ITU: intention to use
MEC: modified exposure claim
MRTP: modified risk tobacco product
S-IQ: adult smokers with an intention to quit
S-NIQ: adult smokers with no intention to quit
SP: study product
TNP: tobacco and nicotine products
TNU18-24: tobacco and nicotine products never-users aged 18-24 years
TNU25+: tobacco and nicotine products never-users aged 25 years and older
