# Supplementary material for: Effects of a Modified Exposure Claim for an e-Cigarette on Claim Comprehension, Behavioral Intentions, and Risk Perceptions Among US Adult Tobacco Users and Nonusers: Randomized Experimental Study
Source: JMIR Form Res. 2026 Apr 20;10:e85802. doi: 10.2196/85802 (PMC13139832; doi:10.2196/85802)
Supplement: Multimedia Appendix 1 [file formative_v10i1e85802_app1.docx]

**Multimedia Appendix 1**

Effects of a Modified Exposure Claim for an E-Cigarette on Claim Comprehension, Behavioral Intentions, and Risk Perceptions Among U.S. Adult Tobacco Users and Non-Users: A Randomized Experimental Study

**Section 1: Definitions of the 6 Participant Groups**

**Section 2: Study Product Marketing Brochures**

**Section 3: Additional Claim Comprehension Results.**

**Section 4: Participant Disposition.**

**Section 5: Cigarette and e-Cigarette Use Characteristics of Study Groups.**

**Section 6: Modified Exposure Claim (MEC) Comprehension Questions and Response Options.**

## Section 1: Definitions of the 6 Participant Groups

The study included the following 6, mutually exclusive groups:

**Group 1: Smokers with no intention to quit smoking** defined as those who:

- - are above the legal smoking age (21 years of age or older);
  - have smoked at least 100 cigarettes in their lifetime;
  - currently smoke cigarettes for 4 or more days per month (no brand restrictions; disregarding religious fasting); and
  - *do not intend* to quit smoking based on the stages of change (SOC) measure.

**Group 2: Smokers with intention to quit smoking** defined as those who:

- - are above the legal smoking age (21 years of age or older);
  - have smoked at least 100 cigarettes in their lifetime;
  - currently smoke cigarettes for 4 or more days per month (no brand restrictions; disregarding religious fasting); and
  - *intend* to quit smoking based on the SOC measure.

**Group 3: E-cigarette users** defined as those who:

- - are above the legal smoking age (21 years of age or older);
  - have used an e-cigarette at least 100 times in their lifetime; and
  - currently use e-cigarettes daily. Note, Adult e-cigarette Users may include participants who otherwise qualify for groups 1, 2, and 4.

**Group 4: Former smokers** defined as those who:

- - are above the legal smoking age (21 years of age or older);
  - have quit cigarette smoking for at least 30 consecutive days prior to the time of their enrollment; and
  - have smoked at least 100 cigarettes in their lifetime and have smoked at least 1 cigarette per day.

**Group 5: TNP never-users (aged 18-24)** defined as those who:

- - are at least 18 years of age and under the age of 25;
  - have never been a user of any nicotine or tobacco-containing products at all OR:
    1. have never been a regular user (i.e., daily) of cigarettes and e-cigarettes; and
    2. have smoked less than 100 cigarettes in their lifetime; and
    3. have used e-cigarettes less than 100 times in their lifetime; and
    4. have never used other tobacco or nicotine-containing products consistently (i.e., with some type of regularity, e.g., every day, a few times every week).

**Group 6: TNP never-users (aged 25+)** defined as those who:

- - are 25 years of age or above;
  - have never been a user of any nicotine or tobacco-containing products at all OR:
    1. have never been a regular user (i.e., daily) of cigarettes and e-cigarettes; and
    2. have smoked less than 100 cigarettes in their lifetime; and
    3. have used e-cigarettes less than 100 times in their lifetime; and
    4. have never used other tobacco or nicotine-containing products consistently (i.e., with some type of regularity, e.g., every day, a few times every week).

The smoking-related classifications in the groups above were determined based on participants’ self-reported smoking/using status, consistent with guidelines established by the World Health Organization [38].

## Section 2: Study Product Marketing Brochures

**(A)**

**(B)**


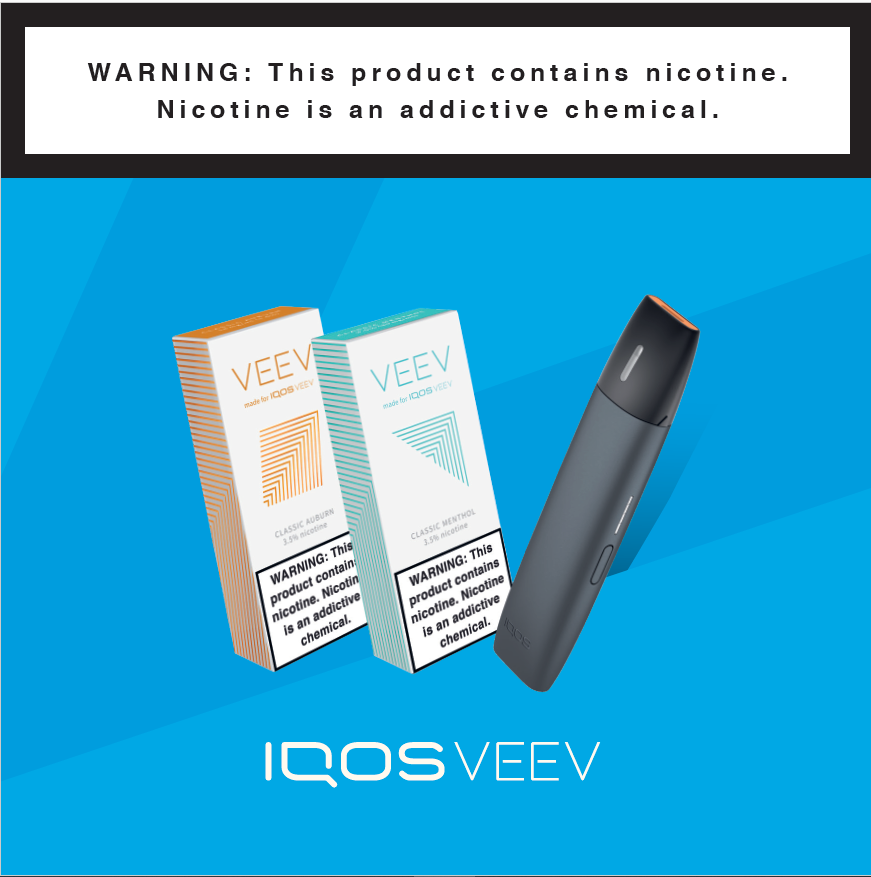

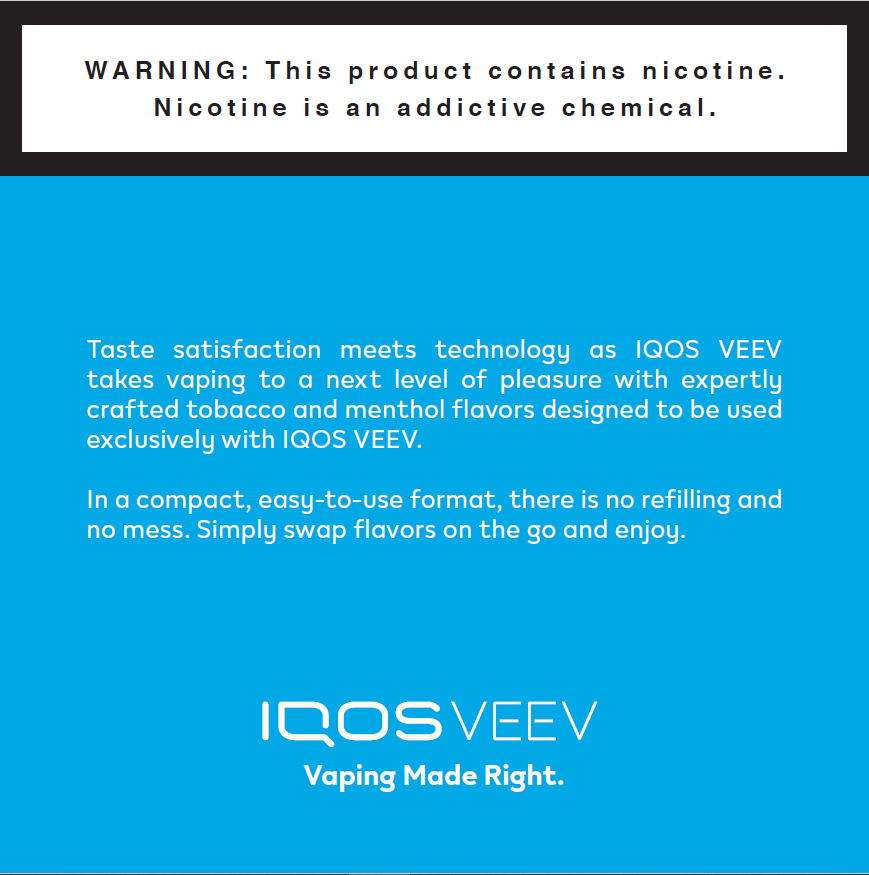

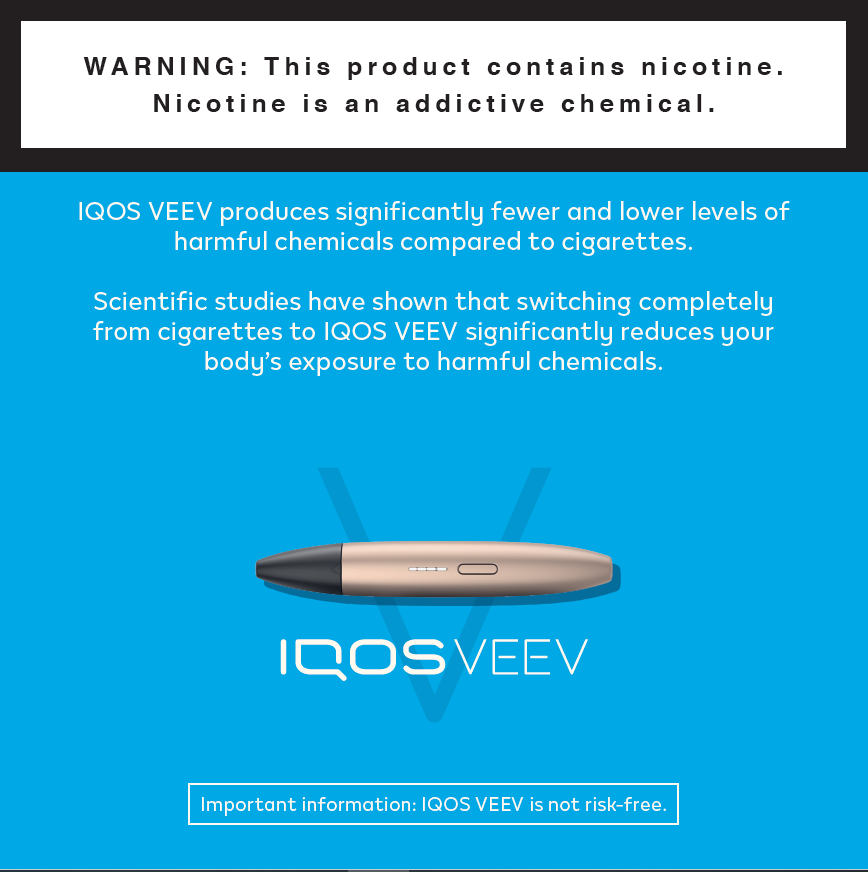

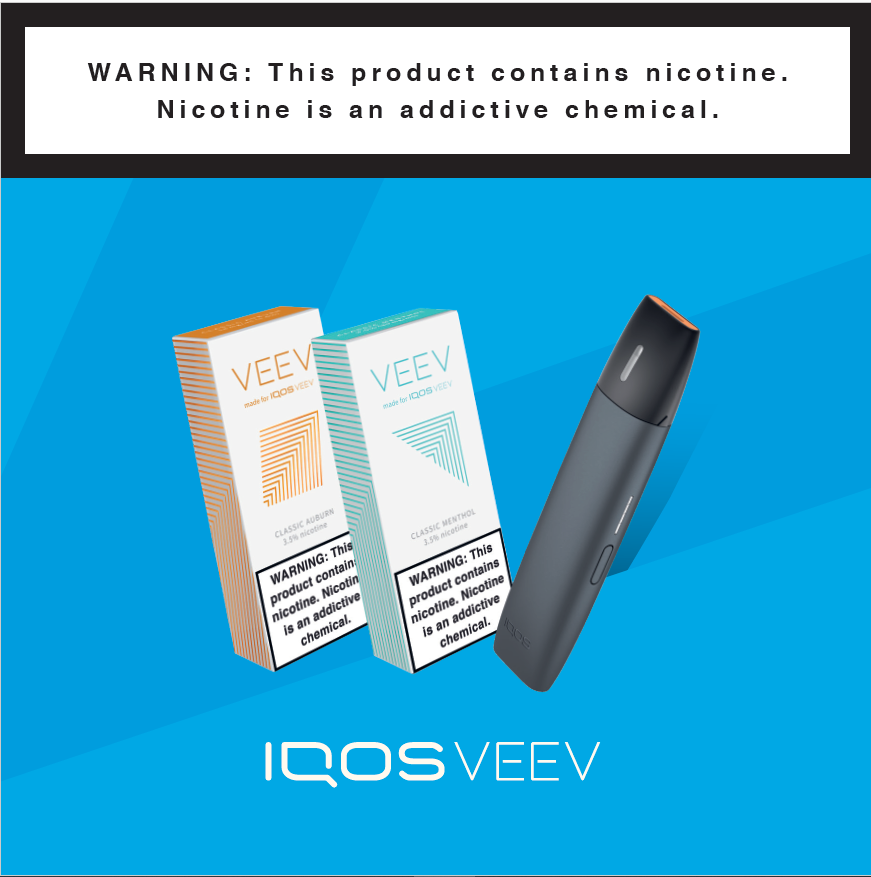

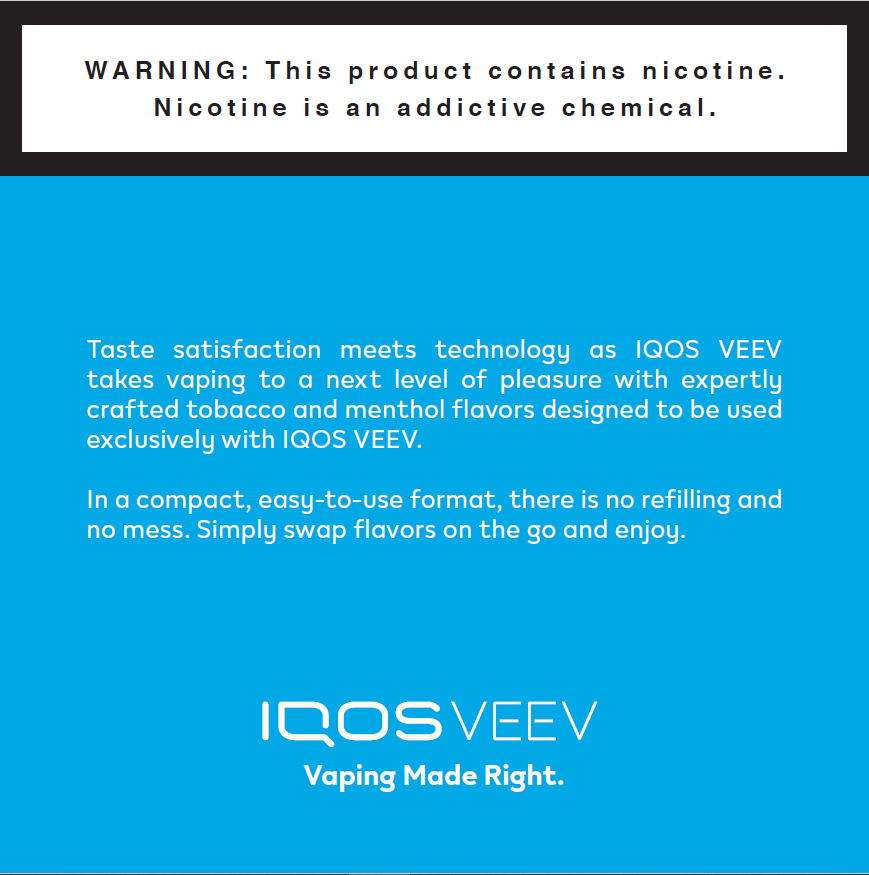

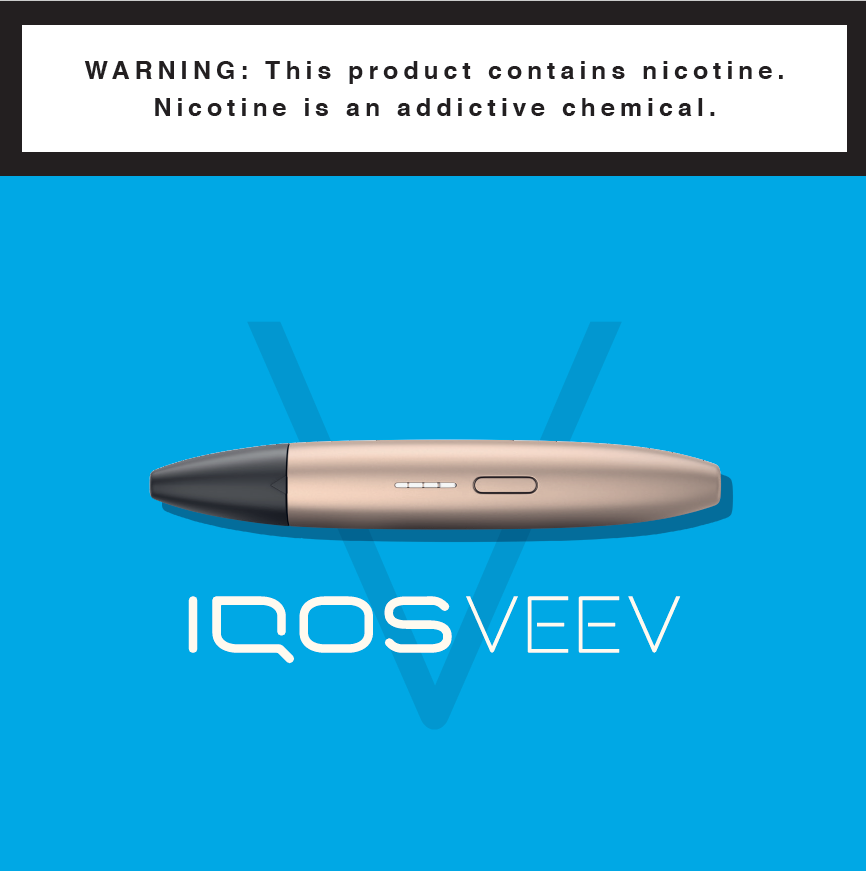


Figure S1. Marketing brochures for the Study Product (SP) used in a U.S. based, randomized, online experimental study. Panel (A) shows the brochure shared with participants in the Test condition, which has the modified exposure claim (MEC) on page 3. Panel (B) shows the brochure shared with participants in the Control condition, which is identical to the brochure in Panel A except for the omission of the MEC on page 3. The brochures consisted of three pages and were presented to adult participants from 6 predefined tobacco‑use groups. Study enrollment and data collection occurred between 14 November 2022 and 14 April 2023.

##

## Section 3: Additional Claim Comprehension Results

Table S1. Comprehension of key communication messages in the Study Product marketing brochure among U.S. adults participating in a randomized, online experimental study. Participants were randomized to view a product brochure either with (Test) or without (Control) a modified exposure claim for the Study Product (an e-cigarette). Data are stratified by 6 mutually exclusive tobacco‑use groups and by study condition. Participants were recruited across 4 U.S. regions. Study enrollment and data collection occurred between 14 November 2022 and 14 April 2023.

| Assessment | Statistic | S-NIQ | | S-IQ | | ECU | | FS | | TNU18-24 | | TNU25+ | | Total | |  |
| --- | --- | --- | --- | --- | --- | --- | --- | --- | --- | --- | --- | --- | --- | --- | --- | --- |
|  |  | Control | Test | Control | Test | Control | Test | Control | Test | Control | Test | Control | Test | Control | Test |  |
|  | N* | 299 | 307 | 301 | 299 | 314 | 315 | 307 | 312 | 330 | 318 | 374 | 375 | 1925 | 1926 |  |
| Understanding that SP contains nicotine: *"According to the material, does the IQOS VEEV product contain nicotine?"* | | | | | | | | | | | | | | | | |
| Yes (correct) | n (%) | 295 (98.7) | 301 (98.0) | 296 (98.3) | 291 (97.3) | 313 (99.7) | 305 (96.8) | 304 (99.0) | 305 (97.8) | 329 (99.7) | 313 (98.4) | 370 (98.9) | 371 (98.9) | 1907 (99.1) | 1886 (97.9) |  |
| No | n (%) | 4 (1.3) | 3 (1.0) | 5 (1.7) | 8 (2.7) | 1 (0.3) | 9 (2.9) | 2 (0.7) | 6 (1.9) | 1 (0.3) | 5 (1.6) | 4 (1.1) | 4 (1.1) | 17 (0.9) | 35 (1.8) |  |
| Don't know / not sure | n (%) | 0 (0.0) | 3 (1.0) | 0 (0.0) | 0 (0.0) | 0 (0.0) | 1 (0.3) | 1 (0.3) | 1 (0.3) | 0 (0.0) | 0 (0.0) | 0 (0.0) | 0 (0.0) | 1 (0.1) | 5 (0.3) |  |
| Missing | n | 0 | 0 | 0 | 0 | 1 | 0 | 0 | 0 | 0 | 0 | 0 | 0 | 1 | 0 |  |

| Assessment | Statistic | S-NIQ | | S-IQ | | ECU | | FS | | TNU18-24 | | TNU25+ | | Total | |  |
| --- | --- | --- | --- | --- | --- | --- | --- | --- | --- | --- | --- | --- | --- | --- | --- | --- |
|  |  | Control | Test | Control | Test | Control | Test | Control | Test | Control | Test | Control | Test | Control | Test |  |
| Understanding that SP is a vaping product: *"According to the material, what kind of product is IQOS VEEV?"* | | | | | | | | | | | | | | | | |
| A vaping product / an electronic cigarette (correct) | n (%) | 283 (94.6) | 294 (95.8) | 294 (97.7) | 291 (97.3) | 295 (93.9) | 295 (93.7) | 295 (96.1) | 299 (95.8) | 322 (97.6) | 303 (95.3) | 354 (94.7) | 348 (92.8) | 1843 (95.7) | 1830 (95.0) |  |
| Other | n (%) | 14 (4.7) | 10 (3.3) | 7 (2.3) | 7 (2.3) | 15 (4.8) | 18 (5.7) | 12 (3.9) | 11 (3.5) | 8 (2.4) | 14 (4.4) | 17 (4.5) | 23 (6.1) | 73 (3.8) | 83 (4.3) |  |
| Don't know / not sure | n (%) | 2 (0.7) | 3 (1.0) | 0 (0.0) | 1 (0.3) | 4 (1.3) | 2 (0.6) | 0 (0.0) | 2 (0.6) | 0 (0.0) | 1 (0.3) | 3 (0.8) | 4 (1.1) | 9 (0.5) | 13 (0.7) |  |
| Missing | n | 0 | 0 | 0 | 0 | 1 | 0 | 0 | 0 | 0 | 0 | 0 | 0 | 1 | 0 |  |
|  | | | | | | | | | | | | | | | | |

Abbreviations: N*, Number of participants who answered all questions required for the evaluation of this endpoint and were asked the respective question depending on group; Percentages are based on the number in N*; SP, Study Product. S-NIQ = Adult Smokers with No Intention to Quit; S-IQ = Adult Smokers with an Intention to Quit; ECU = Adult E-Cigarette Users; FS = Adult Former Smokers; TNU18-24; TNP Never-Users aged 18 to 24 years; TNU25+ = TNP Never-Users aged 25 years and older.

## Section 4: Participant Disposition.
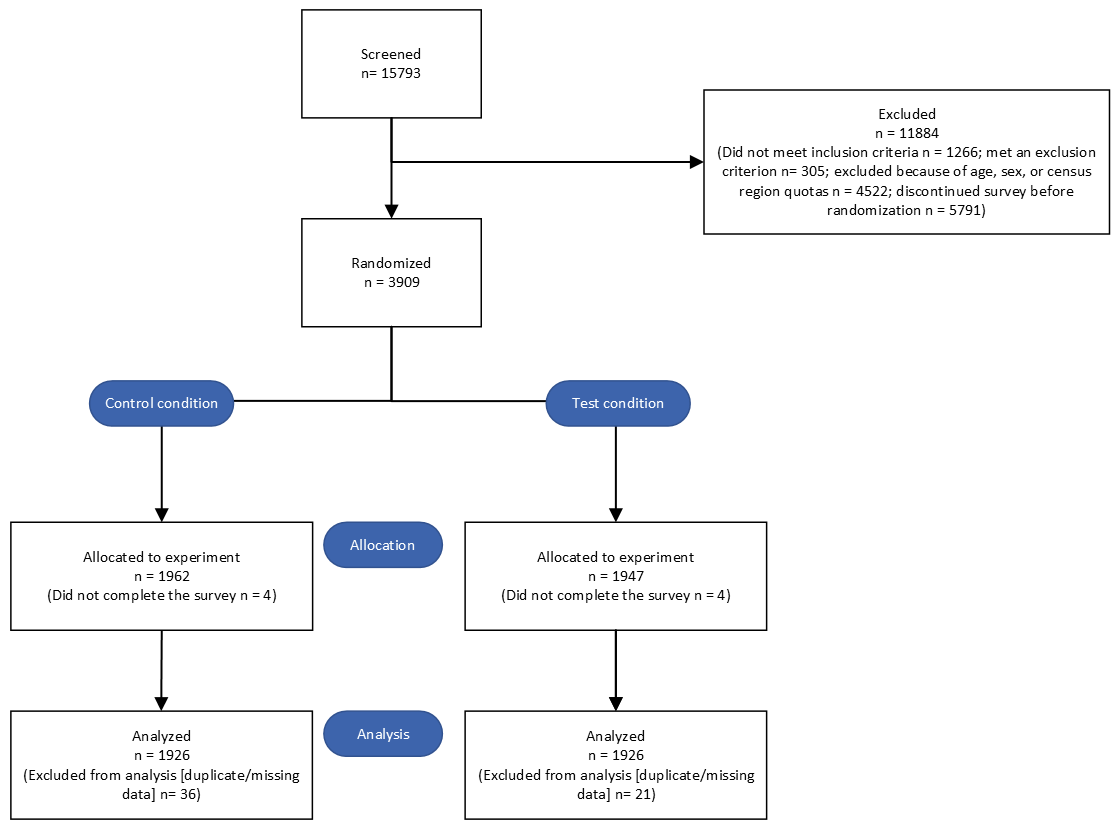


Figure S2. CONSORT (Consolidated Standards of Reporting Trials) flow diagram for a U.S. based, randomized, online experimental study evaluating a modified exposure claim for the Study Product (an e-cigarette). Diagram shows the numbers of participants screened, excluded, randomized, and included in analyses across Test and Control conditions. Study enrollment and data collection occurred between 14 November 2022 and 14 April 2023.

## Section 5: Cigarette and e-Cigarette Use Characteristics of Study Groups

Table S2. Cigarette and e‑cigarette use characteristics of U.S. adults participating in a randomized, online experimental study evaluating a modified exposure claim for the Study Product (an e-cigarette). Participants were randomized to view a product brochure either with (Test) or without (Control) a modified exposure claim. Data are shown for 4 of the 6 mutually exclusive tobacco‑use groups and stratified by study condition. Study enrollment and data collection occurred across the United States between 14 November 2022 and 14 April 2023.

| Variable | Statistic | SNIQ | | S-IQ | | ECU | | FS | |
| --- | --- | --- | --- | --- | --- | --- | --- | --- | --- |
|  |  | Control | Test | Control | Test | Control | Test | Control | Test |
|  | N* | **299** | **307** | **301** | **299** | **315** | **315** | **307** | **312** |
| Cig. smoking frequency |  |  |  |  |  |  |  |  |  |
| Daily | n (%) | 204 (68.2) | 212 (69.1) | 218 (72.4) | 200 (66.9) | - | - | - | - |
| Occasionally | n (%) | **95 (31.8)** | **95 (30.9)** | **83 (27.6)** | **99 (33.1)** | - | - | - | - |
| Smoking days in past month (occasional smokers) | M (SD) | 10.7 (8.6) | 9.9 (7.7) | 10.7 (7.9) | 10.0 (8.5) | - | - | - | - |
| Number of cigarettes smoked per smoking day | M (SD) | 13.3 (7.9) | 11.7 (5.9) | 13.3 (6.1) | 13.1 (6.9) | 11.6 (7.5) | 14.2 (8.3) | - | - |
| When first smoked |  |  |  |  |  |  |  |  |  |
| Within past 12 months | n (%) | 4 (1.3) | 8 (2.6) | 16 (5.3) | 11 (3.7) | 263 (97.8) | 9 (3.2) | - | - |
| ≥ 12 months ago | n (%) | 295 (98.7) | 299 (97.4) | 285 (94.7) | 288 (96.3) | 263 (97.8) | 269 (96.8) | - | - |
| When last smoked |  |  |  |  |  |  |  |  |  |
| Within past 12 months | n (%) | - | - | - | - | - | - | 23 (7.5) | 30 (9.6) |
| ≥ 12 months ago | n (%) | - | - | - | - | - | - | 284 (92.5) | 282 (90.4) |
| EC use occasions per day | M (SD) | - | - | - | - | 17.1 (19.6) | 15.2 (18.0) | - | - |
| When first used an EC |  |  |  |  |  |  |  |  |  |
| Within past 12 months | n (%) | - | - | - | - | 47 (14.9) | 31 (9.8) | - | - |
| ≥ 12 months ago | n (%) | - | - | - | - | 268 (85.1) | 284 (90.2) | - | - |
|  |  |  |  |  |  |  |  |  |  |

Abbreviations: N, number of participants; M, mean; SD, standard deviation; S-NIQ = Adult Smokers with No Intention to Quit; S-IQ = Adult Smokers with an Intention to Quit; ECU = Adult E-Cigarette Users; FS = Adult Former Smokers;.

## Section 6: Modified Exposure Claim (MEC) Comprehension Questions and Response Options

**Understanding that *IQOS VEEV* produces significantly lower levels of harmful chemicals compared to cigarettes.**

“According to the material, what should Kelly say when asked which of the following statements best describes the amount of harmful chemicals produced by *IQOS VEEV* compared to cigarettes? Should Kelly say that…”

1. *IQOS VEEV* produces higher levels of harmful chemicals than cigarettes
2. *IQOS VEEV* produces the same levels of harmful chemicals as cigarettes
3. *IQOS VEEV* produces lower levels of harmful chemicals than cigarettes [Correct]
4. *IQOS VEEV* does not produce harmful chemicals
5. Don’t know/not sure

**Understanding that the exposure to harmful chemicals is significantly reduced.**

“According to the material, what should Jennifer say when asked which of the following statements best describes the exposure to harmful chemicals when switching completely from cigarettes to *IQOS VEEV*? Should Jennifer say that the exposure to harmful chemicals …”

1. Is significantly increased
2. Is significantly reduced [Correct]
3. Is eliminated
4. Is the same as continuing to smoke cigarettes
5. Don’t know/not sure

**Understanding that the exposure to harmful chemicals is significantly reduced when switching completely from cigarettes to *IQOS VEEV*.**

“The material states that the exposure to harmful chemicals is significantly reduced when switching completely from cigarettes to *IQOS VEEV*. What should Greg, who is currently smoking cigarettes, do to reduce his body’s exposure to harmful chemicals? Should Greg…”

1. Continue to smoke the same number of cigarettes and use *IQOS VEEV*
2. Reduce the number of cigarettes and use *IQOS VEEV*
3. Stop smoking cigarettes and only use *IQOS VEEV* [Correct]
4. Increase the number of cigarettes and use *IQOS VEEV*
5. Don’t know/not sure

**Understanding that using *IQOS VEEV* is not risk-free.**

“According to the material, is there any risk associated with using *IQOS VEEV*? ”

1. Yes [Correct]
2. No
3. Don’t know/not sure
